# Correction: Seasonal influenza: Modelling approaches to capture immunity propagation

**DOI:** 10.1371/journal.pcbi.1012213

**Published:** 2024-06-13

**Authors:** Edward M. Hill, Stavros Petrou, Simon de Lusignan, Ivelina Yonova, Matt J. Keeling

There is an error in Fig 3. The row labels for rows 4 and 5 were ordered incorrectly: the label for row 4 should read ‘B/Victoria’ and the label for row 5 should read ‘B/Yamagata’. Please see the correct Fig 3 here.

**Fig 3:**
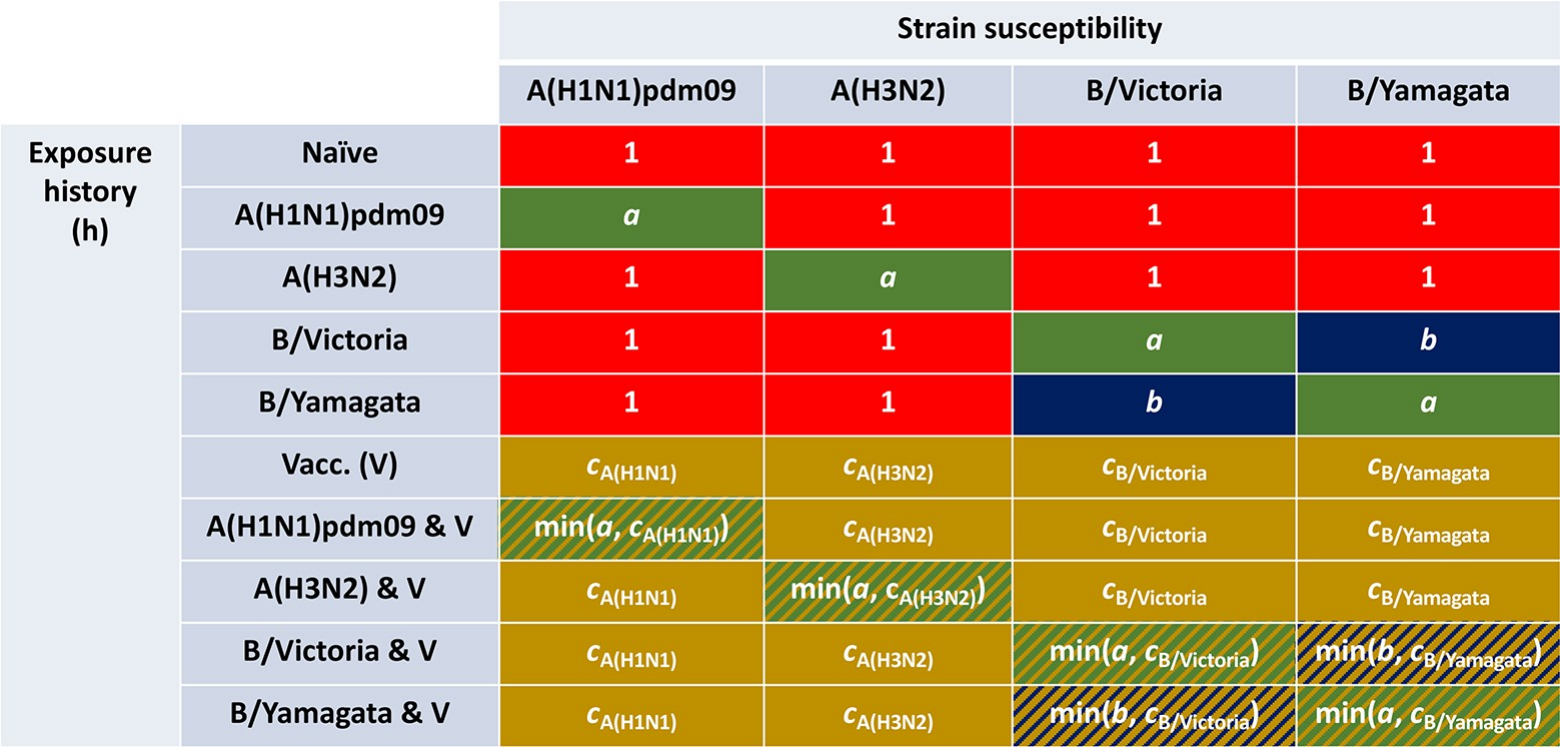
Infographic presenting the interaction between exposure history and susceptibility. The interaction between exposure history *h* and susceptibility to strain *m* in the current influenza season, *f*(*h*, *m*), was classified into ten distinct groups: One group for the naive (uninfected and not vaccinated, row 1); one group per strain, infected but not vaccinated (rows 2–5); one group for those vaccinated and experiencing no natural infection (row 6); one group per strain for being infected and vaccinated (rows 7–10). We let *a* denote modified susceptibility to strain *m* given infection by a strain *m* type virus the previous influenza season (dark green shading), *b* modified susceptibility due to cross-reactivity between type B influenza lineages (dark blue shading), and *c*_*m*_ the change in susceptibility to strain m given vaccination in the previous influenza season (gold shading). Unmodified susceptibilities retained a value of 1 (red shading). We enforced 0 < *a*,*b*,*c*_*m*_ < 1.
